# Supplementary figures and images for: A Qualitative PCR Assay for the Discrimination of Bubaline Herpesvirus 1, Bovine Herpesvirus 1 and Bovine Herpesvirus 5
Source: Microorganisms. 2023 Feb 24;11(3):577. doi: 10.3390/microorganisms11030577 (PMC10056083; doi:10.3390/microorganisms11030577)

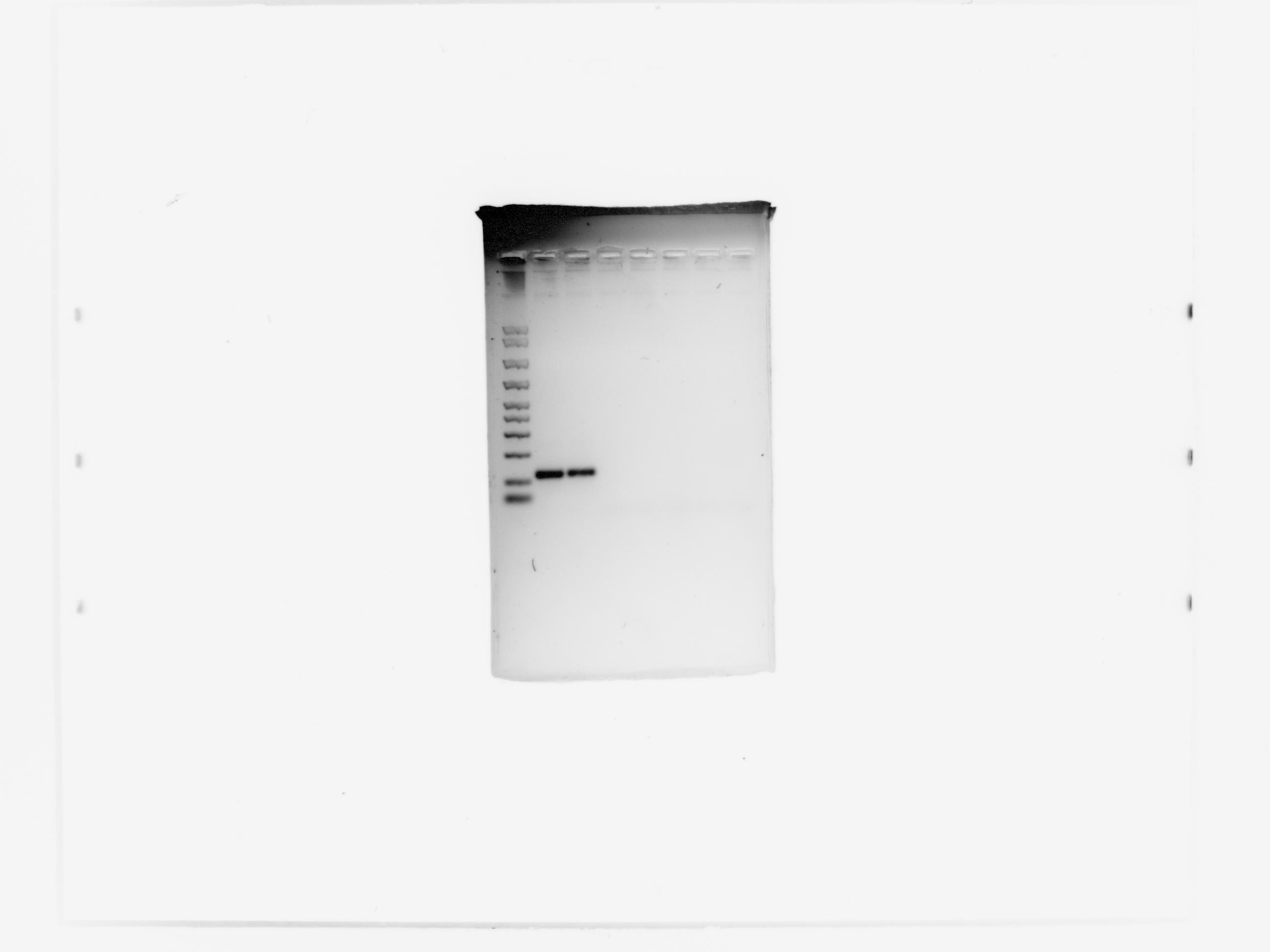

Supplement: Supplementary file 1 [file microorganisms-11-00577-s001.zip › supplemental figures/Figure S1.jpg]

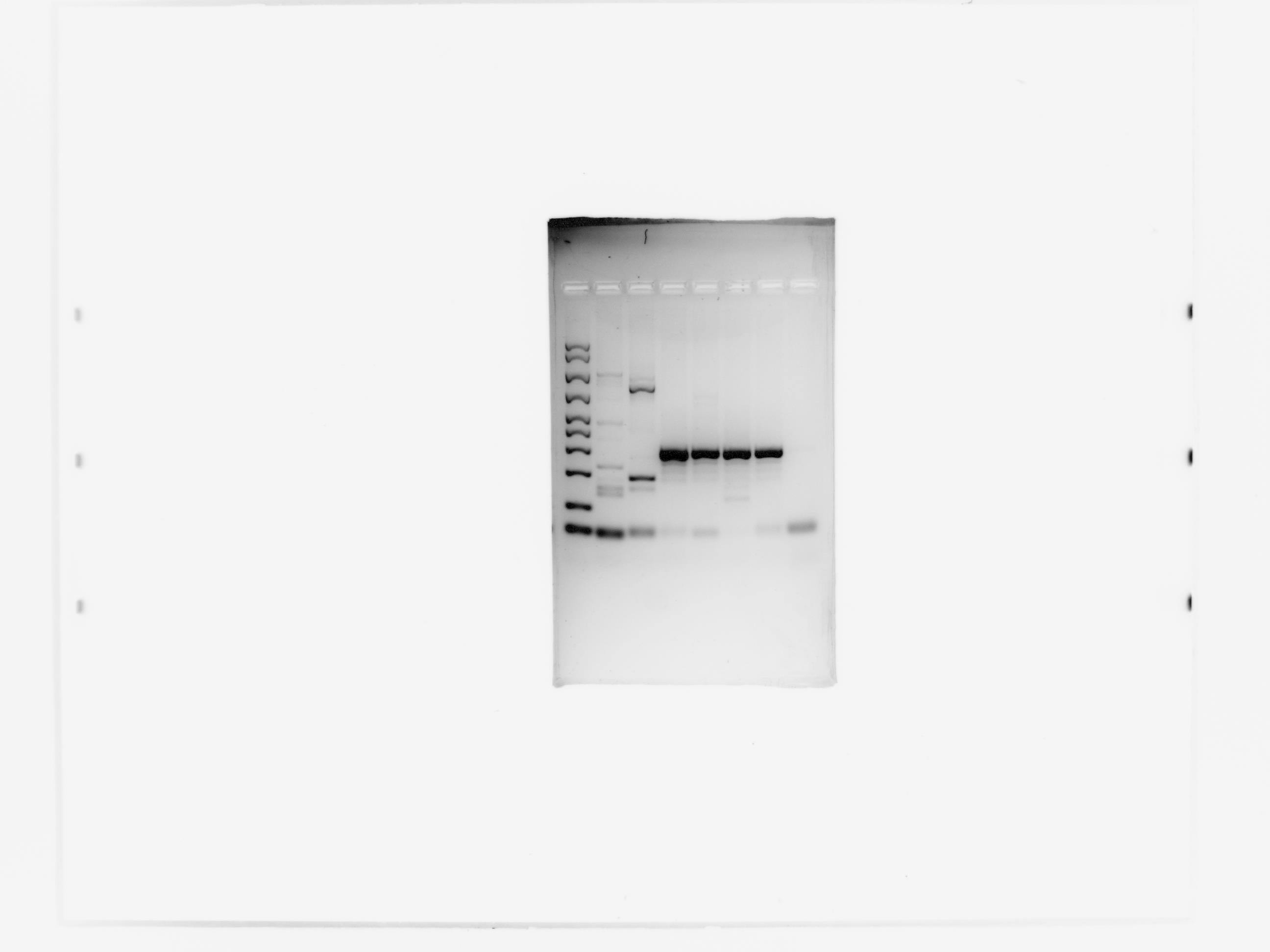

Supplement: Supplementary file 1 [file microorganisms-11-00577-s001.zip › supplemental figures/Figure S2.jpg]

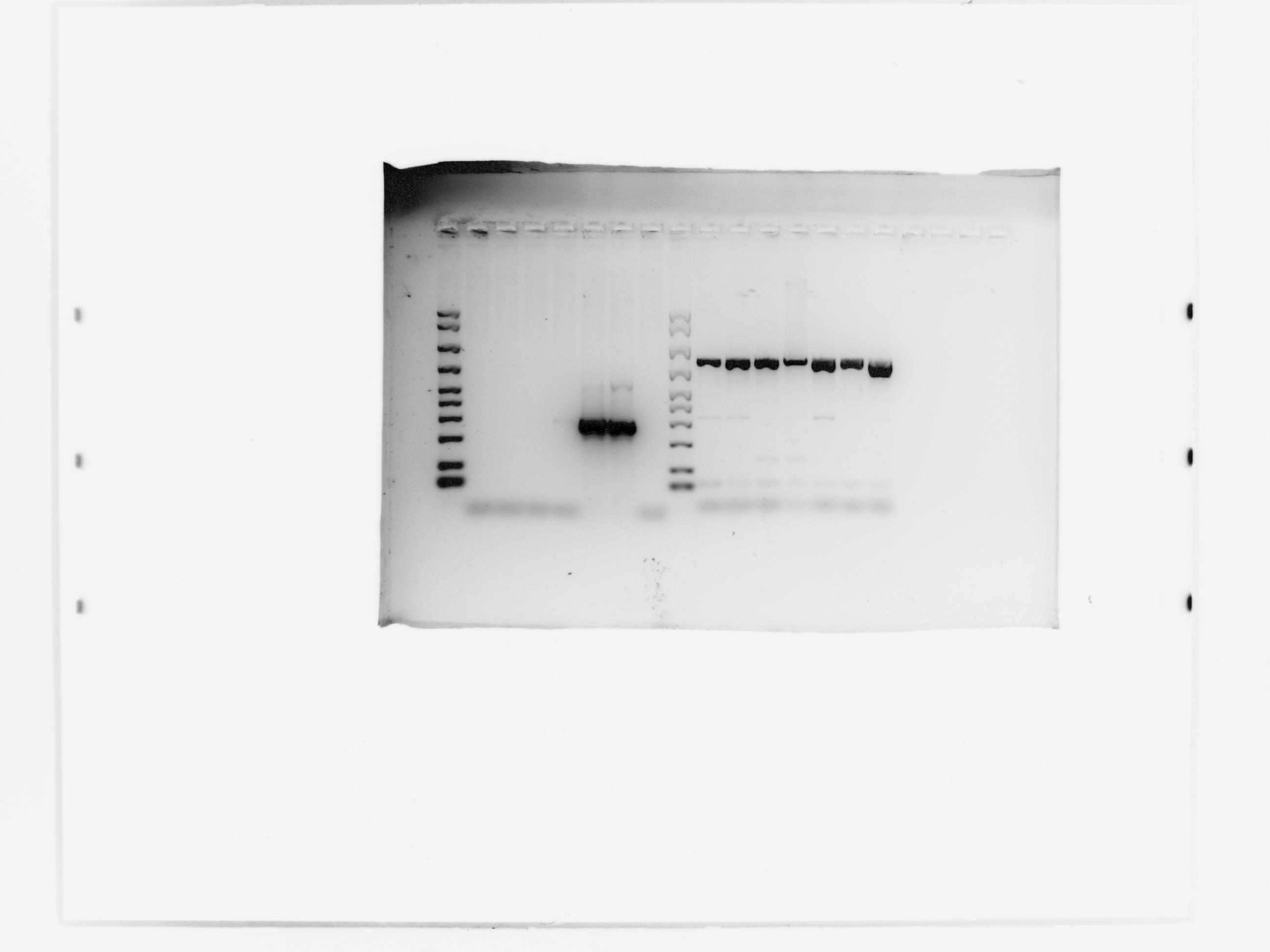

Supplement: Supplementary file 1 [file microorganisms-11-00577-s001.zip › supplemental figures/Figure S3.jpg]
